# A phase I study of MLN4924 and belinostat in relapsed/refractory acute myeloid leukemia or myelodysplastic syndrome

**DOI:** 10.1007/s00280-024-04742-9

**Published:** 2025-01-17

**Authors:** Keri R. Maher, Danielle Shafer, Dale Schaar, Dipankar Bandyopadhyay, Xiaoyan Deng, John Wright, Richard Piekarz, Michelle A. Rudek, R. Donald Harvey, Steven Grant

**Affiliations:** 1 Massey Comprehensive Cancer Center, Virginia Commonwealth University, Richmond, VA USA; 2 Inova Schar Cancer Center, Fairfax, VA USA; 3 Rutgers Cancer Institute of New Jersey, New Brunswick, NJ USA; 4 Cancer Therapy and Evaluation Program, National Cancer Institute, Baltimore, MD USA; 5 The Sidney Kimmel Comprehensive Cancer Center at Johns Hopkins University, Baltimore, MD USA; 6 Department of Oncology, School of Medicine, Johns Hopkins University, 1650 Orleans Street, Baltimore, MD USA; 7 Division of Clinical Pharmacology, Department of Medicine, School of Medicine, Johns Hopkins University, Baltimore, MD USA; 8 Department of Hematology and Medical Oncology, Emory University School of Medicine, Atlanta, GA USA; 9 Department of Pharmacology and Chemical Biology, Emory University School of Medicine, Atlanta, GA USA

**Keywords:** Acute myeloid leukemia, Myelodysplastic syndrome, Pevonedistat, Belinostat

## Abstract

**Purpose:**

Relapsed and/or refractory acute myeloid leukemia and high-risk myelodysplastic syndrome continue to have a poor prognosis with limited treatment options despite advancements in rational combination and targeted therapies. Belinostat (an HDAC inhibitor) and Pevonedistat (a NEDD8 inhibitor) have each been independently studied in hematologic malignancies and have tolerable safety profiles with limited single-agent activity. Preclinical studies in AML cell lines and primary AML cells show the combination to be highly synergistic, particularly in high-risk phenotypes such as p53 mutant and FLT-3-ITD positive cells. Here, we present the safety, pharmacokinetics and pharmacodynamics of belinostat and pevonedistat in a dose escalation Phase I study in AML and High-Risk MDS.

**Methods:**

Eighteen patients (16 with AML, 2 with MDS) were treated at 5 dose levels (belinostat 800–1000 mg/m^2^, pevonedistat 20–50 mg/m^2^). Safety and tolerability were assessed according to protocol defined dose limiting toxicities (DLTs). Correlative pharmacokinetic and pharmacodynamic analyses were performed.

**Results:**

No dose limiting toxicities were noted. Most Grade 3 or 4 toxicities were hematologic in nature. The best response was stable disease in four patients, and complete remission in one patient who qualified as an exceptional responder. Pharmakokinetic studies revealed no association between drug exposure and best response. Pharmacodynamic RT-PCR studies demonstrated post-treatment increases in several proteins, including quantitative increases in the oxidative stress protein NQO1, ferroptosis protein SLC7A11, and GSR, linked to glutathione metabolism and oxidative stress, as did the anti-oxidants SRXN1 and TXNRD1.

**Conclusions:**

Patterns of post-treatment changes in correlative pharmacodynamic parameters may suggest possible mechanistic changes in the DNA damage response, oxidative damage, and ferroptosis pathways. The combination of pevonedistat plus belinosat is safe in an adult relapsed and/or refractory AML/High-Risk MDS population with modest but notable activity in this heavily treated, high risk population. Our findings also raise the possibility that certain extremely poor prognosis AML patients may respond to a regimen combining two targeted agents that have little or no activity when administered individually.

**Trial registration:**

ClinicalTrials.gov ID NCT03772925, first posted 12/12/2018; CTEP Identifier 10246.

## Introduction

Despite considerable progress in elucidating the underlying biological mechanisms of myeloid leukemogenesis, Acute Myeloid Leukemia (AML) continues to have a poor prognosis in adults [1, 2], particularly for those with relapsed or refractory disease, with 5 year overall survival of approximately 10% [3–5]. Extensive efforts are underway to improve these outcomes with pathophysiologically rational combination strategies targeting relevant cellular pathways.

Belinostat is a Histone Deacetylase Inhibitor (HDACi) which exerts multiple biologic effects in cancer cells, including induction of apoptosis, cell cycle arrest, and autophagic cell death [6–9]. HDACis act by modifying chromatin structure, leading to an open architecture that promotes the expression of genes associated with cell differentiation and apoptosis [10]. As malignant cells express higher levels of HDAC than their normal counterparts, the latter are relatively protected from HDACi induced DNA damage and cell death [11, 12]. Belinostat has established anti-cancer activity in a variety of tumors, including having an FDA-indication for relapsed/refractory peripheral T-cell lymphoma [13]. It also has been shown to exert single-agent activity in relapsed/refractory AML and myelodysplastic syndrome with some patients displaying stable disease over several months [14, 15]. Combination therapy with belinostat and either azacitidine or bortezomib in advanced myeloid malignancies including AML and MDS has yielded clinically meaningful results in a limited number of patients based upon reports of early phase trials involving heavily pre-treated patients [16, 17].

Pevonedistat is a first-in-class small molecule inhibitor of the NEDD8-activating enzyme (NAE): a component of the NEDD8 conjugation pathway regulating apoptosis via inhibition of the ubiquitin–proteasome system [18]. Preclinical studies of pevonedistat in mouse xenograft models of solid tumors, lymphoma, and AML have demonstrated inhibition of tumor growth, raising the possibility that this new class of agents may be of benefit in a variety of malignancies [19–22]. In the clinical setting, pevonedistat has shown modest single-agent activity in AML with, 17% complete remission (CR) + partial remission (PR) with a Maximum Tolerated Dose of 59 mg/m^2^ on a D1,3,5 dosing schedule in a 21-day cycle [23], and a non-statistically significant improvement in survival (23.8 vs 20.6mos) in combination with azacitidine in a subset of patients with high-risk MDS in a post-hoc analysis using a substantially lower dose of 20 mg/m^2^ D1,3,5 [24]. The optimal combinatorial dosing of pevonedistat with other agents is unknown.

Multiple biological considerations support the concept that combined NAE inhibition and HDAC inhibition could be effective in relapsed/refractory AML. For example, by blocking the degradation of IκBα, pevonedistat triggers NF-κB inhibition, which antagonizes myeloid leukemia-initiating cell survival and disables an HDACi compensatory cytoprotective response in AML cells [19, 25]. Both of these agents induce oxidative injury and DNA damage in leukemic cells [8, 19, 26], the latter by promoting accumulation of the DNA licensing factor CDT1 and inducing DNA re-replication [27]. Moreover, HDACis may reciprocally increase pevonedostat anti-leukemic activity by blocking DNA damage responses e.g., homologous recombination and non-homologous end-joining, as well as by disrupting DNA damage checkpoints [28, 29]. Preclinical studies in AML cell lines and primary AML cells show the combination of pevonedistat and belinostat to be highly synergistic, particularly in high-risk phenotypes such as p53 mutant and FLT-3-ITD positive cells [30]. Moreover, a regimen combining pevonedistat and belinostat was well tolerated in NSG AML xenograft models, while significantly improving survival compared to single-agent treatment [30]. Together, these concepts provide a preclinical rationale for this therapeutic combination.

In the present study we assessed the safety and tolerability of pevonedistat combined with belinostat in patients with relapsed/refractory AML or myelodysplastic syndrome, and report toxicities, activity, and pharmacodynamic interactions. The primary objective was determination of the maximum tolerated dose (MTD)/Recommended Phase 2 Dose (PR2D) for pevonedistat with belinostat. Secondary objectives included description of toxicities, analysis of anti-leukemic activity/responses, pharmacokinetics, and assessment of correlative studies characterizing pharmacodynamic interactions between these agents.

## Materials and methods

### Drug supply

Pevonedistat HCL (MLN4924) was supplied by Takeda. Belinostat (PXD101) was supplied by Spectrum Pharmaceuticals. Both products were distributed by Pharmaceutical Management Branch of National Cancer Institute’s (NCI) Cancer Therapy Evaluation Program (CTEP) andprepared, stored, and administered according to manufacturer’s guidelines.

### Study design and participants

This was a phase I multi-center single arm study dose escalation study in a 3 + 3 design of pevonedistat and belinostat in R/R AML or MDS. This NCI Experimental Therapeutics Clinical Trials Network study (NCT03772925) was approved by the Massey Cancer Center Protocol Review committee and Central Human Subjects Review Board. All subjects signed IRB- approved informed consent forms. Eighteen patients were enrolled at two sites in the United States. Patients were > 18 years old with ECOG performance status < or = 2 with adequate organ function based on liver and renal function tests, without an uncontrolled coagulopathy/bleeding disorder, uncontrolled hypertension, baseline QTc prolongation, significant cardiopulmonary disease or arrhythmia, and not having received antineoplastic therapy within 14 days of study drug (except cytoreductive hydroxyurea).

Belinostat was administered by a 30 min intravenous (IV) infusion on days 1 through 5 of each cycle; pevonedistat was administered by a 60 min IV infusion on days 1, 3 and 5 immediately after belinostat. The treatments were repeated on 21-day cycles until disease progression or unacceptable toxicity. The dose escalation schedule is described in Table 1. The regimen was administered on an outpatient basis.

**Table 1:** Dose escalation schedule

Dose level	Belinostat		MLN4G24 (pevonedistat)	
	Dose (mg/m^2^/day)	Administration and schedule	Dose (mg/m^2^/day)	Administration and schedule
− 1	800	Intravenously over 30 min once daily on days 1–5 of each 21-day cycle	15	Intravenously over 60 min once daily on days 1, 3, and 5 of each 21-day cycle^b^
1^a^	800		20	
2	800		25	
3	800		37	
4	1000		37	
5	1000		50	

^a^Starting dose level

^b^When both drugs are given on the same day (Days 1, 3, 5), start the MLN4624 (pevonedistat) infusion within 60 min after the completion of belinostat infusion

### Outcomes

Safety and tolerability were assessed according to protocol defined dose limiting toxicities (DLTs). All adverse events (AEs) were characterized and reported according to the NCI Common Terminology Criteria for Adverse Events (CTCAE) version 5.0. Patients who were not evaluable for DLT were replaced.

Bone marrow (BM) aspiration was performed every 2 cycles for treatment response, which was classified using the International Working Group for AML and European Leukemia Net (ELN) to include complete remission (CR), complete remission with incomplete blood count recovery CRi, partial response (PR), stable disease (SD), and progressive disease (PD) [31].

### Correlative pharmacokinetic studies

Blood samples for measurement of plasma concentrations were collected on day 1 of cycle 1 for both belinostat and pevonedistat. Belinostat, PK samples were collected pretreatment, 5 min prior to the end of the infusion, and at 0.25, 0.5, 1, 2, 4, 6, 8, and 24 h after the end of the infusion. Pevonedistat, PK samples were collected pretreatment and 0.5, 1, 2, 2.5, 5, and 24 h after the start of the infusion. Plasma was stored at − 80 °C until analysis. Concentrations of pevonedistat were quantified over the range of 1 to 500 ng/mL using a validated liquid chromatography-tandem mass spectrometry (LC/MS/MS) method at Tandem Labs (West Trenton, NJ) [32]. Concentrations of belinostat were determined over the range of 30–5000 ng/mL with dilutions of up to 1:100 v/v using a validated LC/MS/MS method at the Analytical Pharmacology Core Laboratory at the Sidney Kimmel Comprehensive Cancer Center at Johns Hopkins [33].

Pharmacokinetic parameters were calculated from individual concentration–time data using standard noncompartmental methods as implemented in Phoenix WinNonlin version 8.2 (Pharsight A Certara Company, Cary, North Carolina). Pharmacokinetic parameters were summarized using descriptive statistics. Differences between the pharmacokinetic parameters between dose levels or BSA-based dose were evaluated statistically via the nonparametric Kruskal–Wallis test. All statistical tests were performed using JMP Statistical Discovery software (version 7; SAS Institute, Cary, NC).

### Correlative pharmacodynamic studies

Pharmacodynamic studies were performed on samples from consenting patients with ≥ 40% blasts in the peripheral blood for RT-PCR studies. Alternatively, a flow cytometric assay involving cell permeabilization and intracellular staining of proteins [34] was used to analyze samples consisting of ≥ 10% blasts. In the first case, RNA was isolated at baseline (pre-) and 6 h post treatment. RT-qPCR relative expression levels of various genes was then analyzed. Values for each gene were normalized to the average expression of the endogenous reference genes. Results of analysis were displayed as the mean relative level of assayed target genes ± standard deviations.

Samples were also collected from consenting patients for analysis of changes in protein expression. Specimens were obtained prior to and 24 h (± 6 h) following treatment. Peripheral blood mononuclear cells were isolated from whole blood samples using Ficoll-Hypaque according to the manufacturer’s protocol, and subsequently cryopreserved at − 80 °C. For flow cytometric analysis, a previously described method applicable for AML specimens was employed as previously described with minor modifications [34]. Isolated cells were fixed and permeabilized with True/Phos Perm Buffer (Biolegend), and stained with PE Cy7 conjugated CD45, APC-CD3, and APC Cy7-CD20 antibodies (Biolegend) in conjunction with PE-Bim, PE-p-Chk1, PE-p-cdc2 Y15, FITC-p-cdc2 T14, FITC-ˠH2AX (all Cell Signaling) and FITC-p-HH3 (Biolegend) antibodies as well as the appropriate isocontrols. Cells were analyzed using a BD FACSCanto flow cytometer. Analysis of biomarkers was conducted on the gated CD45dim/low CD3-CD20- leukemic cell population. The mean fluorescence intensity ratio of signal to isocontrol for pretreatment samples for each specimen was set at 100%.

## Results

### Safety and efficacy measures

Eighteen patients were treated at five dose levels (belinostat 800–1000 mg/m^2^, pevonedistat 20–50 mg/m^2^). Thirty-three percent were male, with a median age of 67.5 (range 41–74), 16 of 18 patients had R/R AML (89%), 2 had Myelodysplastic Syndrome (11%) (Table 2). Median number of prior lines of therapy was 3 (range 1–11). Three patients were enrolled and evaluable for DLT in the first of three dose levels. Four subjects were enrolled at dose level four, with one non-evaluable, and five subjects were enrolled at dose level 5 with two non-evaluable. Notably, no dose limiting toxicities were encountered at any dose level.

**Table 2:** Baseline characteristics

Category	Treated patients^a^ *n* (%)
Age [Year, Mean (SD)]	63.7 (12.1)
Gender	
Female	12 (66.7%)
Male	6 (33.3%)
Performance status (ECOG)	
0	4 (22%)
1	14 (78%)
Race	
Black of African American	6 (33%)
White	10 (55.6%)
Unknown	2 (11%)
Ethnicity	
Hispanic or Latino	2 (11.1%)
Not Hispanic or Latino	15 (83.3%)
Unknown	1 (5.6%)
Disease histology	
Acute myeloid leukemia	16 (89%)
Myelodysplastic syndrome	2 (11%)

^a^The denominator for the percentage is 18 (number of treated patients)

Six patients (33%) experienced one or more grade 3 or greater AEs likely attributable to the study drugs. Excluding electrolyte abnormalities, these toxicities were hematologic in nature (Table 3). Twenty-two percent of patients experienced a decrease in WBC count; 17% of patients experienced a decrease in neutrophil count; 28% of patients had a decrease in platelet counts; 17% anemia; 6% neutropenic fever.

**Table 3:** Number of patients with high grade (> = 3) related adverse events

Toxicity^a^	Grade 3 *n* (%)	Grade 4 *n* (%)	Total *n* (%)
Anemia	3 (16.7)	0 (0.0)	3 (16.7)
Febrile neutropenia	1 (5.6)	0 (0.0)	1 (5.6)
Fatigue	1 (5.6)	0 (0.0)	1 (5.6)
Neutrophil count decreased	1 (5.6)	2 (11.1)	3 (16.7)
Platelet count decreased	1 (5.6)	4 (22.2)	5 (27.8)
White blood cell decreased	3 (16.7)	1 (5.6)	4 (22.2)

^a^When a particular patient had the same toxicity on more than one occasion, only the highest grade is listed

The maximum number of cycles reached was 8. A CR was achievedin one patient (6%) (on Dose Level 4), who was considered an exceptional responder.. Four patients had stable disease (22%).

Progressive disease was noted in the remaining patients. The most common reason for treatment discontinuation was disease progression 12 (67%), with 2 (11%) discontinuing due to alternative therapy pursued, and 1 (6%) due to side effects, lung infection, attribution unrelated to study drugs.

### Pharmacokinetics, pharmacodynamics, and correlative studies

Belinostat and pevonedistat pharmacokinetic data were available for 16 and 11 patients, respectively (Table 4). Belinostat, belinostat glucuronide, and pevonedistat pharmacokinetic parameters were not different by dose level. When assessing by BSA-based dose, belinostat clearance (p = 0.05) was significantly higher at 1000 mg/m^2^ compared with 800 mg/m^2^. Pevonedistat AUC_INF_ (p = 0.02) was significantly higher at 37 mg/m^2^ compared with 20 or 25 mg/m^2^_._ There were no associations between exposure and best response or reason for discontinuation of study.

**Table 4:** Plasma pharmacokinetic parameters

Belinostat	Dose (mg/m^2^)	^preEOI C^max (µM)	^T^max (h)	^AUC^INF (µM·h)	^T^1/2 (h)	Cl (L/hr)
Dose level 1	800	109.0, 144.5 (2)	0.4, 0.5 (2)	54.6, 75.2 (2)	0.59, 0.67 (2)	60.1, 87.5 (2)
Dose level 2	800	119.6 ± 50.3 (3)	0.5 (0.4–0.5; 3)	64.7 ± 27.4 (3)	1.36 ± 0.61 (3)	83.1 ± 30.1 (3)
Dose level 3	800	124.9 ± 35.0 (3)	0.4 (0.4–0.5; 3)	61.0, 87.4 (2)	1.12, 1.46 (2)	61.8, 82.4 (2)
	800	123.4 ± 34.3 (8)	0.4 (0.4–0.5; 8)	67.5 ± 19.1 (7)	1.13 ± 0.50 (7)	77.3 ± 20.7 (7)
Dose level 4	1000	96.5 ± 46.0 (4)	0.4 (0.3–0.6; 4)	60.3 ± 39.2 (3)	1.02 ± 0.44 (3)	121.2 ± 60.2 (3)
Dose level 5	1000	113.7 ± 29.4 (3)	0.4 (0.4–0.4, 3)	45.1 ± 13.4 (4)	0.79 ± 0.12 (4)	138.0 ± 29.1 (4)
	1000	103.9 ± 37.8 (7)	0.4 (0.3–0.6; 7)	51.6 ± 25.8 (7)	0.89 ± 0.30 (7)	130.8 ± 41.4 (7)

Belinostat glucuronide	Dose (mg/m^2^)	^preEOI C^max (µM)	^T^max (h)	^AUC^INF (µM·h)	^T^1/2 (h)
Dose level 1	800	178.4, 246.7 (2)	0.4, 0.8 (2)	489.8, 733.6 (2)	3.42, 3.69 (2)
Dose level 2	800	141.9 ± 18.8 (3)	0.8 (0.4–0.8; 3)	380.3 ± 127.2 (3)	4.29 ± 0.68 (3)
Dose level 3	800	144.2 ± 30.0 (3)	0.8 (0.7–0.8; 3)	488.0, 702.3 (2)	3.04, 4.23 (2)
	800	160.5 ± 41.5 (8)	0.8 (0.4–0.8; 8)	507.8 ± 168.6 (7)	3.89 ± 0.65 (7)
Dose level 4	1000	214.0 ± 86.8 (4)	0.8 (0.3–0.9; 4)	647.4 ± 390.3 (4)	3.87 ± 0.91 (4)
Dose level 5	1000	193.0 ± 18.8 (4)	0.7 (0.4–0.8; 4)	624.2 ± 165.3 (4)	4.15 ± 0.83 (4)
	1000	203.5 ± 59.2 (8)	0.8 (0.3–0.9; 8)	635.8 ± 277.7 (8)	4.01 ± 0.82 (8)

Pevonedistat	Dose (mg/m^2^)	^preEOI C^max (nM)	^T^max (h)	^AUC^INF (nM·h)	^T^1/2 (h)	Cl (L/hr)
Dose level 1	20	278, 311 (2)	0.4, 0.6 (2)	2282, 2850 (2)	5.89, 6.12 (2)	28.5, 37.6 (2)
Dose level 2	25	372 ± 93.2 (3)	0.6 (0.4–1.0; 3)	2952 ± 666 (3)	6.52 ± 1.38 (3)	37.5 ± 3.4 (3)
Dose level 3	37	897 ± 26.0 (3)	0.9 (0.9–1.0; 3)	4949 ± 426 (3)	5.97 ± 0.34 (3)	34.9 ± 2.9 (3)
Dose level 4	37	841 ± 455 (3)	1.0 (0.6–1.0; 3)	4601 ± 1423 (3)	5.61 ± 1.50 (3)	35.5 ± 8.3 (3)
	37	869 ± 290 (6)	0.9 (0.6–1.0; 6)	4775 ± 958 (6)	5.79 ± 0.99 (6)	35.2 ± 5.6 (6)
Dose level 5	50	N.A	N.A	N.A	N.A	N.A

Data are presented in the table as mean±SD^(n)^. ^T^max is presented as median (range; n). If n<3, the actual values are reported

^*AUC*^*INF* area under the plasma concentration–time curve to infinity, Cl systemic clearance, ^*C*^*max* peak plasma concentration, *NA* not available, Tmax time to peak concentration, ^*T*^*1/2* half−life

**Fig. 1:**
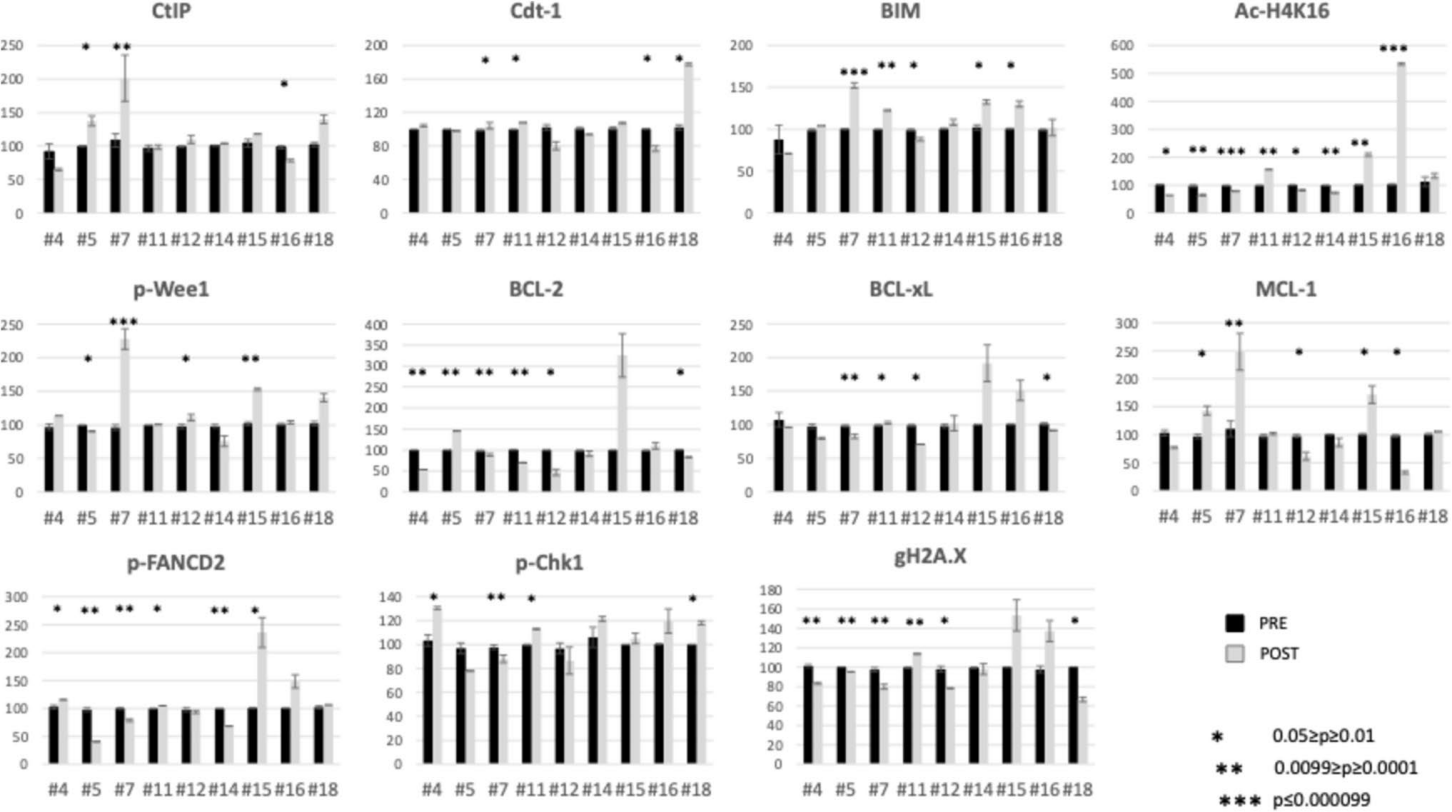
Flow cytometry analysis of pre- and post-treatment patient samples. Peripheral blood mononuclear cells (PBMNCs) were isolated at baseline (Pre) and 24 h after treatment (Post). PBMNCs for each sample were analyzed for levels of CtIP, Cdt-1, BIM, Ac-H4K16, p-Wee1, BCL-2, BCL-xL, MCL-1, p-FANCD2, p-Chk1 and gH2AX. Analysis of biomarkers were performed on the CD45^dim^ SS^low^ CD3- CD20- population. The Mean fluorescence Intensity (MFI) ratios of biomarker signals to their isocontrols for pre-treatment samples for each patient was set as 100%. Analysis for each individual biomarker is represented as a bar chart showing the relative level of the assayed protein in samples after averaging, including standard deviation bars. The Student t test was performed to indicate the significance of changes in post versus pre-treatment samples

Pharmacodynamic studies employing a flow cytometric assay [34] monitoring intracellular protein levels were performed on peripheral blood leukemic specimens from 9 patients obtained prior to therapy and 24 h after administration of the first dose of pevonedistat. The goal was to determine if changes in DNA damage response proteins observed pre-clinically following pevonedistat/belinostat exposure would be recapitulated in patient cells after in vivo exposure to these agents. Figure 1 represents responses seen in intracellular protein levels. Responses appear variable, with no clear trends elucidated in the evaluated biomarkers, although a relatively consistent increase in BIM was seen in many of the patients analyzed. BIM, a member of the BCL-2 family, is important in apoptotic pathways, and treatment with chemotherapy has previously been identified as inducing BIM [35].

**Fig. 2:**
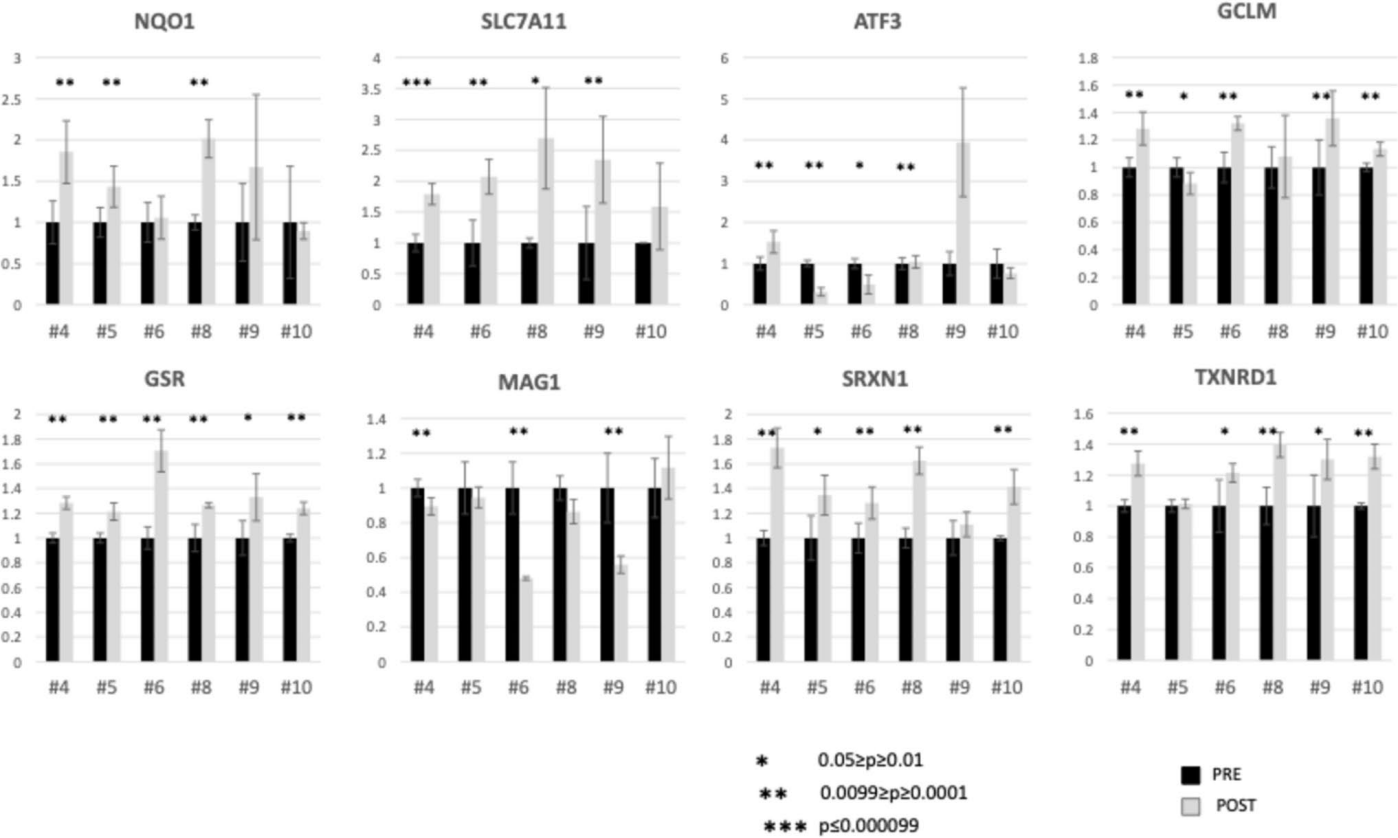
RT-PCR analysis of pre- and post-treatment patient samples. RNA was isolated from cells at baseline (Pre) and 6 h after treatment (Post). RT-qPCR analysis was performed to determine the relative expression levels of NQO1, SLCA11, ATF3, GCLM, GSR, MAG1, SRXN1 and TXNRD1. The expression of each gene was normalized to the average expression levels of the 4 endogenous reference genes e.g., 18S, B2M, RPLP0 and UBC. Analysis of each individual biomarker is shown as a bar chart illustrating the relative level of the assayed target gene, including standard deviation bars. The Student t test was performed to show the significance of post- versus pre-treatment changes

RT-PCR studies performed on samples obtained from 6 patients demonstrated consistent post-treatment increases in several proteins tested (Fig. 2). For example, quantitative increases in the oxidative stress protein NQO1 in all but one patient. Statistically significant increases in SLC7A11, a protein associated with ferroptosis, were noted in all but one sample [36, 37]. GSR, linked to glutathione metabolism and oxidative stress [38] increased in all specimens assayed. Similarly, the anti-oxidants SRXN1 and TXNRD1 increased in 5 specimens analyzed.

In summary, results of pharmacodynamic studies monitoring post-treatment protein expression were variable. Trends warranting further analysis have emerged, including those in ferroptosis, glutathione metabolism, and oxidative stress pathways that warrant further exploration. Unfortunately, the exceptional responder did not consent to these studies, thus, conclusions regarding the response patterns of responders could not be drawn.

Underlying leukemic cytogenetic and molecular mutations were not available for most patients, however, the exceptional responder’s AML signature included Trisomy 8, and BRAF V600E and TET2 mutations. A larger sample size would be necessary to determine if any correlates between disease characteristics, Pharmacodynamic, and clinical responses to this regimen exist.

## Discussion

This study is the first combination of a NEDD 8 inihibitor and an HDAC inhibitor in the treatment of relapsed/refractory AML or High Risk MDS. The results of this study indicate that the combination of pevonedistat plus belinostat is safe in adult patients with relapsed/refractory AML or high-grade myelodysplastic syndrome. It confers a manageable, primarily hematologic toxicity profile, with a modest activity in this heavily pre-treated population. This indicates that a subset of patients with extremely poor prognostic features may respond to a regimen combining agents with modest (pevonedistat) [23] or minimal (belinostat) [13] single-agent activity. Notably, there was potentially increased signal for anti-leukemic activity as dose levels progressed. Two patients at dose level 3 achieved stable disease and underwent four and eight cycles of therapy, respectively. The patient achieving CR (primary refractory AML with TET 2 and Trisomy 8) was treated at dose level 4. A maximum tolerated dose was not reached and no DLTs were noted at the highest dose level, which was significantly higher than previous combination therapy studies e.g., with 5-azacytidine [24]. Maximum tolerated dose, while a common Phase I endpoint, may not be as clinically relevant as optimal biologic dose (OBD), particularly in targeted agents [39]. Correlative PD studies, such as those included herein, are critical for efforts at determining OBD rather than MTD and can provide background information for future studies with OBD endpoints using either of these agents.

It was not possible to correlate pharmacodynamics features of the regimen with clinical activity given low CR rate. However, the observed patterns of post-treatment changes in pharmacodynamic parameters suggest that for such agents, perturbations in the DDR [40, 41], oxidative damage pathways [38], and potentially ferroptosis [36, 37] represent plausible candidate pharmacodynamic determinants.

As in the case of all such trials employing targeted agents, it is possible that the failure to achieve adequate drug plasma levels might contribute to the limited responses observed. However, it should be noted that pharmacokinetic studies of belinostat [33] and pevonedistat [42] administered at doses similar to those employed here yielded C_max_ levels far in excess of those shown to exert activity in preclinical studies e.g., 100–200 nM [38, 39]. The belinostat, belinostat glucuronide, and pevonedistat exposure were similar to previously reported values [33, 43].

After the Phase III PANTHER trial assessing azacitidine vs azacitidine and pevonedistat in the first line setting for patients with high-risk MDS and AML did not achieve its primary endpoint of event-free survival, development of pevonedistat was halted by the sponsor [24]. While the Panther III trial was substantially different from this study in that it involved a different patient population (treatment-naïve vs relapsed/refractory) as well as a different drug combination (pevonedistat plus 5-azacytidine vs an HDACI), important lessons learned from PANTHER III trial may still apply. For example, a signal for improvement in overall survival in a post-hoc analysis was noted for patients receiving greater than three cycles [24], underscoring the importance of continued treatment with regimens incorporating a hypomethylating agent backbone. Our study is suggestive of similar findings, with CR having been achieved only after four cycles.

## Conclusions

In summary, the results of the present study indicate that a regimen combining the NEDD8 inhibitor pevonedistat with the HDAC inhibitor belinostat is safe and unassociated with DLTs at the dose levels examined. Significantly, a *bona fide* exceptional responder was observed at a dose level (37 mg/m^2^) well below the maximally tolerated pevonedistat dose (59 mg/m^2^) previously shown to exhibit significant single-agent activity. The PK and PD data as presented herein could have important implications for future iterations of this drug in combination in AML. The strategy of enhancing the anti-leukemic activity of this class of agents with HDACIs might be extended to include alternative clinically relevant agents which disrupt the ubiquitin–proteasome system and induce proteotoxic stress. The latter might include agents such as inhibitors of p97 [44] and ubiquitin-activating enzymes (e.g., TAK-243) [45]. Accordingly, preclinical studies designed to test this possibility are currently underway.

## Data Availability

All supporting data and materials will be made available upon reasonable request.
